# *Cyp19a1* (Aromatase) Expression in the *Xenopus* Brain at Different Developmental Stages

**DOI:** 10.1111/jne-12142

**Published:** 2014-04-03

**Authors:** P Coumailleau, O Kah

**Affiliations:** Neuroendocrine Effects of Endocrine Disruptors, IRSET, INSERM U1085, SFR Biosit, Université de Rennes 1Rennes, France

**Keywords:** Aromatase, *Cyp19a1*, radial glia, *Xenopus*, Brain

## Abstract

Cytochrome P450 aromatase (P450arom; aromatase) is a microsomal enzyme involved in the production of endogeneous sex steroids by converting testosterone into oestradiol. Aromatase is the product of the *cyp19a1* gene and plays a crucial role in the sexual differentiation of the brain and in the regulation of reproductive functions. In the brain of mammals and birds, expression of *cyp19a1* has been demonstrated in neuronal populations of the telencephalon and diencephalon. By contrast, a wealth of evidence established that, in teleost fishes, aromatase expression in the brain is restricted to radial glial cells. The present study investigated the precise neuroanatomical distribution of *cyp19a1 mRNA* during brain development in *Xenopus laevis* (late embryonic to juvenile stages). For this purpose, we used *in situ* hybridisation alone or combined with the detection of a proliferative (proliferating cell nuclear antigen), glial (brain lipid binding protein, *Vimentin*) or neuronal (acetylated tubulin; HuC/D; *NeuroβTubulin*) markers. We provide evidence that *cyp19a1* expression in the brain is initiated from the very early larval stage and remains strongly detected until the juvenile and adult stages. At all stages analysed, we found the highest expression of *cyp19a1* in the preoptic area and the hypothalamus compared to the rest of the brain. In these two brain regions, *cyp19a1*-positive cells were never detected in the ventricular layers. Indeed, no co-labelling could be observed with radial glial (brain lipid binding protein, *Vimentin*) or dividing progenitors (proliferating cell nuclear antigen) markers. By contrast, *cyp19a1*-positive cells perfectly matched with the distribution of post-mitotic neurones as shown by the use of specific markers (HuC/D, acetylated tubulin and *NeuroβTubulin*). These data suggest that, similar to that found in other tetrapods, aromatase in the brain of amphibians is found in post-mitotic neurones and not in radial glia as reported in teleosts.

Cytochrome P450 aromatase (P450arom; aromatase), the product of the *cyp19a1* gene, is a microsomal enzyme that converts C19 androgens, such as testosterone or androstenedione, into C18 oestrogens, oestradiol (E_2_) or oestrone, respectively 1–3. Increasing evidence suggests that the production of oestrogens in the vertebrate brain is involved in important physiological and behavioural processes 1,4–6. The best documented function of aromatase (also called oestrogen synthetase) is its role in the organisation of sexually dimorphic structures during development of the hypothalamus in the male rodent 1,7–10. During adult life, such regions will also be activated by local oestrogen synthesis to regulate sexual behaviour 11–13.

The distribution of *cyp19a1* mRNA has been extensively investigated in the brain of mammals and birds 14–16. However, for technical reasons, the use of antibodies in the mammalian brain has proven problematic, whereas, in contrast, aromatase antibodies have been extensively and successfully used in birds 17,18. In mammals and birds, consistent with its roles in the regulation of reproductive and behavioural functions, *cyp19a1*/aromatase is mainly expressed in the hypothalamus, the bed nucleus of the stria terminalis and the amygdala 4–6. In songbirds, aromatase is also involved in the seasonal development and activation of vocal centres 19,20. According to current knowledge, *cyp19a1*/aromatase expression is predominantly in neurones, although expression in astrocytes has been occasionnally reported, particularly after chemical and/or mechanical lesions 21,22. In fish, where two *cyp19a1* genes (*cyp19a1a* and *cyp19a1b)* were identified, a very high levels of aromatase activity was also described in the brain of many teleost species 23–26. Unexpectedly, in teleost fishes, aromatase is only expressed in radial glial cells and its expression is highly dependent upon oestrogens and some aromatisable androgens 23.

In amphibians, aromatase is encoded by a single *cyp19a1* gene leading to two transcripts in the gonads and a single one in the brain. These transcipts differ in their 5-untranslated region but contain an identical open reading frame 27. Importantly, *cyp19a1* transcripts detected in the brain of amphibians encode a biologically active protein 28–30. Reverse transcriptase- polymerase chain reaction (RT-PCR) experiments were performed to study the presence of *cyp19a1* mRNA during brain development 27,31,32. Interestingly, the *cyp19a1* gene was shown to be strongly expressed from early developmental stages and remains at high levels until metamorphosis. In addition, no sex-specific expression of *cyp19a1* gene was observed 27,31,32. The present study provides critical information on the precise sites of expression of *cyp19a1* in the brain of *Xenopus laevis* during development.

To gain more insight into the potential function of aromatase in the brain of amphibians, using *in situ* hybridisation, we have analysed the distribution of *cyp19a1* transcripts in the brain of *X. laevis* from late embryonic to post-metamorphic (juvenile and adult) stages. We provide evidence that the *cyp19a1* gene is expressed very early during brain development and in a region specific manner. In addition, our data indicate that *cyp19a1*-expressing cells comprise exclusively post-mitotic neuronal cells.

## Materials and methods

### Animals and tissue processing

For the present study, 47 South African clawed frogs (*X. laevis*) and two *Xenopus tropicalis* were used. Embryo, larvae, juvenile and adult *Xenopus* were purchased from the CNRS ressource (CRB-UMS3387; http://xenopus.univ-rennes1.fr/). The different developing stages were obtained by *in vitro* fertilisation and maintained in water at 20 °C. Embryo and larvae were staged according to Nieuwkoop and Faber (NF; 1967). Late embryo (NF35), premetamorphic larvae (NF42; NF47; NF49), prometamorphic (NF52; NF58), metamorphic (NF62), post-metamorphic (juveniles/NF66) and adult stages were fixed in PAF4% overnight at 4 °C. Before the fixation procedure, juveniles and adult stages were deeply anaesthetised in a 0.4 mg/ml solution of tricaine methanesulfonate (MS222; Sigma, St Louis, MO, USA), rapidly decapitated and then fixed. The brains were dissected out and postfixed in fresh fixative overnight at 4 °C. After a few washes in phosphate-buffered saline, brains were embedded in paraffin and sectionned transversally at 8 μm into six alternate series of sections. All procedures adhered to the European guidelines (Directive 86/609/EEC). Protocols were approved by the university of Rennes 1 and performed by authorised investigators (Permit number: 75–390).

### *In situ* hybridisation

The cDNA of *Xenopus Vimentin* and *Neuroβtubulin* have been used previously 33,34. For *in situ cyp19a1*, an IMAGE cDNA clone (ThermoScientific, Wilmington, DE, USA; clone ID, 6944814) was used 35. Sense and antisense digoxigenin-labelled riboprobes were transcribed using the Digoxigenin RNA labelling kit in accordance with the manufacturer's instructions (Roche, Mannheim, Germany). The brain sections were processed for *in situ* hybridisation as described previously 36. After revelation using NBT/BCIP substrate, sections were either subjected to immunostaining (see below) or directly counterstained with 4’,6-diamidino-2-phenylindole (DAPI), and mounted in Vectashield medium (Vector Laboratories, Inc., Burlingame, CA, USA). For fluorescent *in situ* hybridisation detection, sections were incubated in HNPP (2-hydroxy-3-naphtoic acid-2′-phenylanilide phosphate)/FastRED solution (Roche) for 2–4 h as described previously 37.

### Immunohistochemistry

To characterise the *cyp19a1*-expressing cells, immunohistochemistry for the brain lipid binding protein (BLBP), proliferating cell nuclear antigen (PCNA), acetylated tubulin (TUB) and HuC/D (HU) was carried out. Following the *in situ* hybridisation procedures, immunofluorescence for BLBP and PCNA was performed as described previously 33. For other immunodetections, we used, under similar conditions, mouse monoclonal antibodies, which recognise acetylated Tubulin (dilution 1 : 100; clone 611B1; Sigma) or HuC/D (dilution 1 : 100; clone 16A11; Invitrogen, Carlsbad, CA, USA) proteins. At the end of the immunodetection procedures, the slides were mounted in Vectashield containing DAPI. As controls, primary or secondary antibodies were omitted.

## Results

### Spatio-temporal expression of the *cyp19a1* gene in the developing brain of *Xenopus*

**Figure 1:**
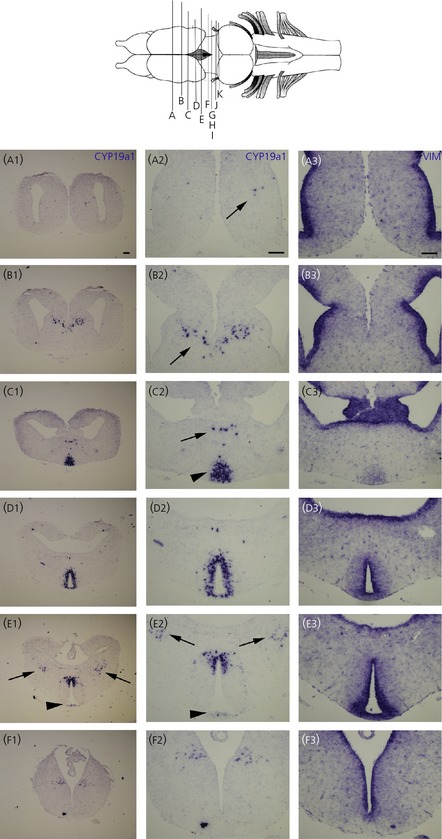
Expression pattern of *cyp19a1* (CYP) (a1–f1 and a2–f2) compared to *Vimentin* (VIM) (a3–f3) in the *Xenopus laevis* prometamorphic larva (NF stage 58) brain. Top: dorsal view of the *X. laevis* brain. Letters correspond to the rostrocaudal location of transverse sections as depicted in the whole brain drawing. Illustrations (a2) to (f2) correspond to higher magnifications of (a1) to (f1) and to adjacent sections of (a3) to (f3). Arrows and arrowheads highlight less conspicuous areas of labelling. For all images, dorsal is to the top. Scale bars = 100 μm.

**Figure 2:**
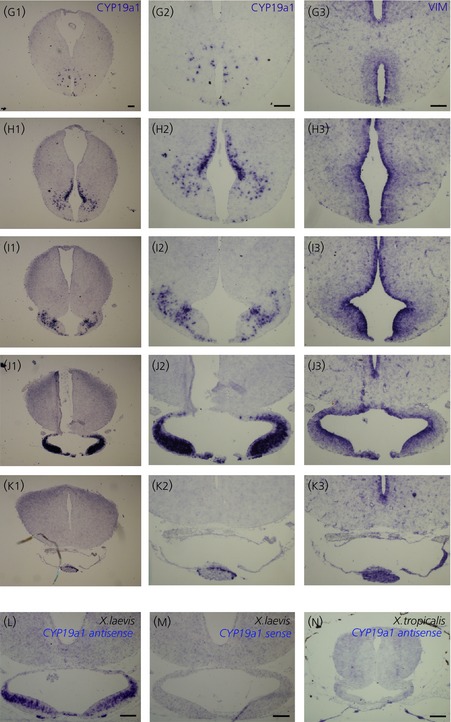
Expression pattern of *cyp19a1* (CYP) (g1–k1 and g2–k2) compared to *Vimentin* (VIM) (g3–k3) in the *Xenopus laevis* prometamorphic larva (NF stage 58) brain. Letters correspond to the rostrocaudal location of transverse sections as depicted in the whole brain drawing of Fig.1. Illustrations (g2) to (k2) correspond to higher magnifications of (g1) to (k1) and to adjacent sections of (g3) to (k3). (l–n) Control *in situ* hybridisation experiments using *cyp19a1* sense (m) or antisense (l and n) probes on *X. laevis* (l and m) or *Xenopus tropicalis* transverse sections. For all images, dorsal is to the top. Scale bars = 100 μm.

The expression of the *cyp19a1* gene was studied by *in situ* hybridisation in the developing brain of *X. laevis* from late embryonic through juvenile stages. Below, we first report the *cyp19a1* expression pattern at the larva prometamorphic stage (Nieuwkoop and Faber stage 58; NF58) from a series of transverse sections through all subdivisions of the brain (Figs1 and 2; n = 3). The nomenclature used in the present study is essentially the same as that employed by D'Amico *et al*. 34. Labelling appeared to be specific because no hybridisation signal was observed with the sense-strand probe (Fig.2l,m). In addition, the conditions used for *in situ* hybridisation were highly stringent because no cross-hybridisation signal could be observed with *cyp19a1* transcripts of *X. tropicalis* (Fig.2n), a close relative of *X. laevis*.

We detected *cyp19a1* transcripts in a variety of telencephalic and diencephalic areas. Rostrally, at mid-telencephalic levels, a hybridisation signal for *cyp19a1* transcripts was first detected in a few cells located in the ventral portion of the septal regions (arrows in Fig.1a1,a2–b1,b2). As shown on adjacent sections and using a *Vimentin* probe, a reliable ventricular marker 33,34,38, the ventricular zone of the septum was devoid of *cyp19a1*-positive cells (compare Fig.1b2,b3). Moving caudally, *cyp19a1*-positive cells were also observed in the bed nucleus of the stria terminalis (arrows in Fig.1c1,c2). However, at this level, the most obvious *cyp19a1*-positive cell population was localised ventrally, just anterior to the preoptic recess (arrowheads in Fig.1c1,c2). Again, no overlapping with *Vimentin* transcripts could be detected (Fig.1c3). In the rostral diencephalon, at the level of the anterior commissure, strong *cyp19a1* expression was detected in the anteroventral preoptic area (Fig.1d1,d2). At this level, a uniform presence of *cyp19a1* transcripts was observed throughout the dorsal and ventral portions close to the preoptic recess. Within the preoptic nucleus, only scattered *cyp19a1*-expressing cells were observable in more lateral areas (Fig.1d1,d2). As highlighted with the *Vimentin* probe on adjacent sections (Fig.1d3), *cyp19a1*-positive cells were observed in large numbers outside the ventricular zone of the preoptic recess (Fig.1d2,d3). *Cyp19a1-*expressing cells were also found in more caudal aspects of the preoptic area, although the staining was restricted to more dorsal regions (Fig.1e1,e2). However, very weak labelling was observed in a compact group of small cells in the most ventral aspect of the preoptic recess of the diencephalic ventricle (i.e. in the organum vasculosum laminae terminalis) (arrowheads in Fig.1e1,e2). On the same transverse section, the *cyp19a1* gene was also significantly expressed more laterally in a group of cells of the amygdala (arrows in Fig.1e1,e2). In the posterior part of the preoptic area, at the level of optic chiasma, the *cyp19a1* gene was expressed moderately in the ventral thalamic region (Fig.1f1,f2). More caudally, the anterior aspect of the infundibular recess was surrounded by scattered *cyp19a1*-positive cells (Fig.2g1,g2). In the mediobasal hypothalamus, *cyp19a1* gene expression was strongly detected at all levels (Fig.2h1,h2–j1,j2). Indeed, *cyp19a1*-positive cells were observed close to the infundibular in its dorsal, middle, basolateral and basal portions, although positive cells were more abundant in the mammilary (Fig.2h2–i2) and tuberal (Fig.2i2–j2) regions. *In situ* hybridisation on diencephalic adjacent sections using the *vimentin* marker again suggest that *cyp19a1*-positive cells are not distributed in the ventricular layer (Fig.2e3–j3).

**Figure 3:**
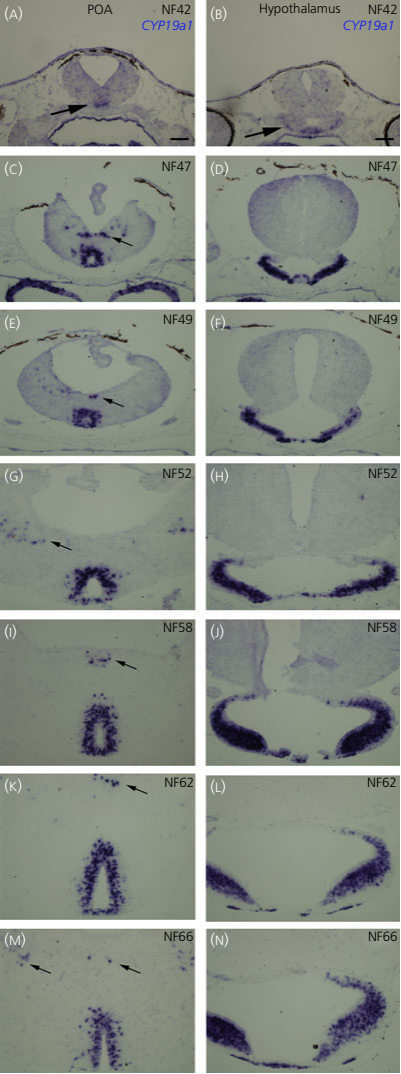
Distribution of *cyp19a1* (CYP) transcripts at different brain developmental stages. *In situ* hybridisations for *cyp19a1* at late embryonic stage 42 (NF42; a, b); at premetamorphic stages NF47 (c, d), NF49 (e, f) and NF52 (g, h); and at prometamorphic NF58 (i, j), metamorphic NF62 (k, l) and post-metamorphic NF66 (juvenile; m, n) stages. For all developmental stages, transverse sections at the levels of the preoptic area (POA) (a, c, e, g, i, k, m) and hypothalamus (b, d, f, h, j, l, n) are shown. Arrows highlight less conspicuous areas of labelling. (l–n) For all images, dorsal is to the top. Scale bars = 100 μm.

In addition to the prometamorphic larva stage (NF58; Figs1 and 2), expression of the *cyp19a1* gene was also investigated at other developmental stages, from late embryonic through juvenile stages (Fig.3; n = 35). *Cyp19a1* expression was never observed in the late embryo (NF35; data not shown) but was detected for the first time in the developing brain at the early larval stage (NF42; Figs3a and 3b). Indeed, two small populations of *cyp19a1*-positive cells were found in the primordia of both preoptic area (arrow in Fig.3a) and hypothalamus (arrow in Fig.3b). Interestingly, at late larval stages or for premetamorphic larvae (NF47, NF49 and NF52), the strongest expression was observed in two regions: the preoptic area (Fig.3c,e,g) and the caudal aspect of the hypothalamus (Fig.3d,f,h). This expression pattern remained similar at later developmental stages, in the prometamorphic (NF58, Fig.3i,j), metamorphic (NF62; Fig.3k,l), juvenile (NF66, Fig.3m,n) and adult (Fig.5d and data not shown) stages. Indeed, across development, the overall pattern of expression did not change dramatically. Messengers appeared in certain brain areas (preoptic and hypothalamic areas) at early larval stages and the signal increased progressively with age in these same areas. In addition, in late larval and post-metamorphic stages, we also found *cyp19a1* expression, although at lower levels, in the other sites (ventral septum, bed nucleus stria terminalis, amygdala and ventral thalamus) described previously for the prometamorphic stage (NF58, Figs1 and 2; arrows in Fig.3; data not shown). It is also interesting to note that, although we studied five animals at each developmental stage, the overall expression pattern of *cyp19a1b* was very consistent from one animal to the other.

**Figure 4:**
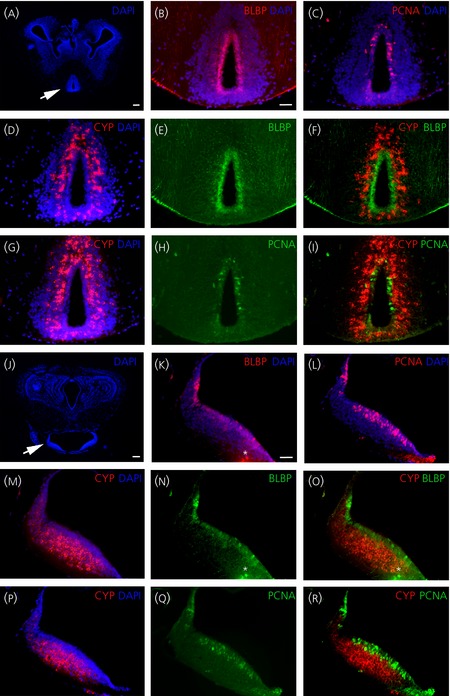
*Cyp19a1*/brain lipid binding protein (BLBP) and *Cyp19a1* (CYP)/proliferating cell nuclear antigen (PCNA) double-stainings in the preoptic and hypothalamic areas of metamorphic (NF62) brain. Transverse sections at the level of the preoptic area (a–i) or hypothalamus (j–r). Arrows in (a) and (j) indicate the precise location of the high magnifications illustrations (b–i) and (k–r), respectively. *In situ* hybridisation using a *cyp19a1* probe (CYP) combined with BLBP (b, e, f, k, n, o) or PCNA (c, h, i, l, q, r) immunohistochemistry. To allow a merge with the 4’,6-diamidino-2-phenylindole (DAPI) staining, colours of BLBP and PCNA immunostainings in (b), (c), (k) and (l) are changed. Asterisks in (k), (n) and (o) indicate background labelling. For all images, dorsal is to the top. Scale bar = 100 μm in (a) and (j); 50 μm in (b–i) and (k–r).

**Figure 5:**
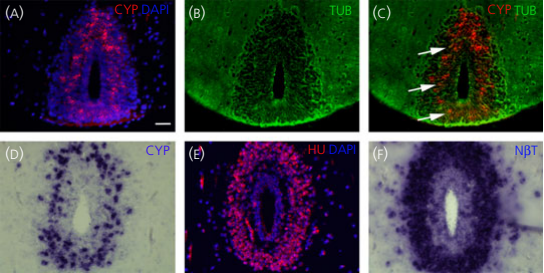
*Cyp19a1* transcripts distribution compared to several neuronal markers on juvenile (a–c) and adult (d–f) preoptic area transverse sections. (a–c) *Cyp19a1* (CYP) *in situ* hybridisations (a, c) combined with acetylated tubulin (TUB) immunodetections (b, c). Arrows indicate some double-stained cells. (d–f) Comparison between *Cyp19a1* (CYP) *in situ* hybridisation (d), HuC/D immunodetection (HU) and *Neuroβtubulin* (NβT) *in situ* hybridisation on adjacent sections. For all images, dorsal is to the top. Scale bar = 50 μm.

### *Cyp19a1* is not expressed in radial glial cells or in mitotic progenitor cells but rather in post-mitotic neurones

*In situ* hybridisation on adjacent sections using the *Vimentin* probe (Figs1 and 2) suggested that *cyp19a1*-positive cells were not localised in the ventricular layers (i.e. in radial glial or in progenitor cells). To firmly demonstrate this point, *in situ* fluorescent hybridisation using the *cyp19a1* antisense probe was combined with immunofluorescence against cytoplasmic BLBP or the PCNA, which are established markers of radial glia or dividing progenitor cells, respectively 33,34. We focused our analysis on the preoptic (arrow in Fig.4a) and caudal hypothalamic (arrow in Fig.4j) areas, comprising regions where *cyp19a1* expression is highest (Figs 1–3; n = 3). As shown in Fig.4, BLBP and PCNA-positive cells were abundant in both the preoptic area (Fig.4b,c,e,h) and hypothalamus (Fig.4k,l,n,q). In addition, as shown in Fig.4(b,e), BLBP antibodies clearly demonstrated the classical morphology of the radial glial cells, i.e. labelling of both cell bodies (in the ventricular layer) and end-feet (at the brain surface). Importantly, no cellular co-localisation between *cyp19a1* and BLBP could be observed in the preoptic area (Fig.4d–f) or in the hypothalamus (Fig.4m–o). In a similar way, there was no colocalisation of PCNA and *cyp19a1*-expressing cells in the preoptic (Fig.4g–i), hypothalamic (Fig.4p–r) and other brain areas with minor sites of *cyp19a1* expression (data not shown), suggesting that *cyp19a1* was not expressed in proliferative ventricular zones. Taken together, these results provided evidence that *cyp19a1* gene is not expressed in radial glial cells and/or in progenitor cells.

Because the above data strongly suggested that *cyp19a1* might be expressed in neuronal cells, we performed *in situ* hybridisation for *cyp19a1* combined with immunocytochemistry against TUB marker, a well known cytoplasmic marker for post-mitotic neurones 39–41. As shown in Fig.5(b), acetyl-Tub (TUB) antibodies produced an intense staining of the axons and a weaker staining of the cell bodies. Unfortunately, double-staining with *cyp19a1* probe and acetyl-Tub antibodies (*CYP*/TUB) demonstrated very few cells positive for both markers (arrows in Fig.5a–c). Indeed, positive double-labelling *CYP*/TUB signals were not entirely convincing as a result of the localisation of *cyp19a1* and acetylated tubulin in different subcellular compartments, with acetylated tubulin proteins being predominantly distributed in axons and *cyp19a1* transcripts in the cell bodies. Therefore, the neuronal marker HU was used because HuC/D proteins are RNA-binding proteins known to shuttle between the nucleus and cytoplasm 42 and immunohistochemistry using HU antibodies displays nuclear and/or peri-nuclear staining 43,44. Because HU immunofluorescence signals did not persist when combined with *in situ* hybridisation conditions, we carefully examined *cyp19a1* and HU labelling on transverse adjacent sections of the preoptic area (Fig.5d,e; n = 3). As shown with the double-staining HU/DAPI (Fig.5e), HuC/D immunostaining was markedly detected in neuronal cell bodies away from the ventricular layer, similar to the localisation of *cyp19a1*-expressing cells (Fig.5d). Using the *NeuroβTubulin* post-mitotic neuronal marker 45 on transverse adjacent sections (Fig.5f), we also detected a similar distribution between *cyp19a1* and *NeuroβTubulin* transcripts (compared Fig.5d,f; n = 3). Importantly, a comparison of *cyp19a1* labelling with neuronal markers (HU and *NeuroβTubulin*) also suggested that *cyp19a1* was not expressed in all neurones but, instead, in sub-populations. Finally, we never observed *cyp19a1* expression in oligodendrocytes, as assessed using the proteolipoprotein marker (data not shown). Taken together, our data demonstrate that the *cyp19a1* gene is expressed in post-mitotic neurones and not in progenitors or radial glial cells.

## Discussion

The present study provides a detailed description of the anatomical distribution of *cyp19a1* transcripts during the development of the central nervous system in *X. laevis*, starting from the late embryonic stage and ending after completion of metamorphosis. We provide evidence that *cyp19a1* gene expression is already significant during brain development in *X. laevis* and presents a regionalised pattern. *Cyp19a1* messengers were first detected at the early laval stage in the primordia of the preoptic and hypothalamic areas and their expression strongly increased, before metamorphosis, at late larval stages. No obvious changes were observed at the prometamorphic, metamorphic and post-metamorphic stages, except that these *cyp19a1*-expressing areas became larger as the brain size increased during development. We also found minor sites of *cyp19a1* expression in the septum, bed nucleus of the stria terminalis, amygdala and ventral thalamus. Previous RT-PCR analyses in *X. laevis* have monitored *cyp19a1* gene expression in the brain from larval stage 48 to juvenile 27,32. Interestingly, PCR-based studies reported a high expression of *cyp19a1* in the brain of *X. laevis* during this time without sex-specific expression. Moreover, our *in situ* hybridisation data in the brain correlate with previous *in vitro* detection of *cyp19a1* expression and/or aromatase enzyme activity in the developing and/or adult brain of other amphibian species, including *X. tropicalis*, *Rana esculenta* and *Pleurodeles waltl* 24,29,31,46,47. Importantly, the present study demonstrates that *cyp19a1* expression appears in specific brain regions at a very early larval stage (NF42), clearly before the sensitive window for sex differentiation of *X. laevis* that occurs between stage 44 and 54 48, and this expression remains elevated until post-metamorphic stages (juvenile and adult). High levels of aromatase activity have been measured in the developing brain of all vertebrate species studied, including mammals 1. Our data confirm that the brain is a major *cyp19a1-*expressing organ in amphibians.

We detected aromatase mRNA in a variety of diencephalic and telencephalic areas, including the medial and lateral septum, bed nucleus of the stria terminalis, amygdala (medial and lateral), preoptic area, ventral thalamus and hypothalamus. Clearly, the major sites of *cyp19a1* expression are within brain regions involved in reproductive functions and behaviour. This expression pattern is well conserved compared to that known in other vertebrate species, notably in birds and mammals, in which *cyp19a1* mRNA and protein have similarly been detected in the medial preoptic area, the ventromedial nucleus of the hypothalamus, bed nucleus stria terminalis and the amygdala (i.e. areas involved in sexual behaviour and neuroendocrine control of reproduction) 14,49,50. Among non-mammalian vertebrates, teleost fishes exhibit exceptionally high levels of aromatase activity in the brain, higher than that in the ovary 51,52, and the functions of such high levels of enzymatic activity still remain open to speculation. Similar to other vertebrates, studies in several different fish species have reported elevated aromatase expression and activity in the telencephalon and diencephalon, notably the preoptic area and caudal hypothalamus 23,53. Expression of *cyp19a1* and/or aromatase activity have also been documented in lizard and snake brains, and its distribution was also correlated with regions that control sex behaviours 54–57. Our results support the idea that the general pattern of *cyp19a1* expression in the adult and developing brain is well conserved among vertebrates, reinforcing the assumption that its functions during development of the brain in amphibians, as well as at the adult stage, are similar to those identified in other vertebrates (i.e in brain sexual differentiation and sexual behaviours). Nevertheless, the broad expression of *cyp19a1* does not exclude important developmental roles other than brain sexual differentiation. In perfect agreement with our study, a large increase in *cyp19a1* gene expression was identified on whole *X. tropicalis* specimens at early developmental stages (NF41) followed by a major aromatase activity at stage NF46 (Langlois *et al*., 2010) and, interestingly, aromatase inhibition at these early developmental stages appears to affect the transcription of genes associated with the thyroid and reproductive axes 47.

In amphibians, two studies have reported distributions for immunoreactive aromatase; data were reported on the distribution of the aromatase protein in the preoptic and mesencephalic tegmentum of adult male *Penaeus esculentus* 58 and in the choroid plexus, olfactory bulbs and paleocortex of *X. laevis* at larva (NF50) stage 59. As noted in the Introduction, there have been a number of technical complications regarding the use of aromatase antisera in mammals, although there has been greater reliability in some non-mammalian vertebrates. These data, based on heterologous antibodies, do not fully match our present results based on a specific *X. laevis* riboprobe. In the anterior preoptic area of *P. esculentus*, few aromatase positive cells were detected in a similar way to the detection of *Xenopus cyp19a1* transcripts. There are several possible explanations for the discrepancies observed among *in situ* hybridisation and immunohistochemical studies, including antisera specificity, different frog species or developmental stages. Future studies that combine both procedures will provide significant advantages.

Although, in mammals and birds, aromatase expression under nomal conditions has been reported mainly in neurones 60–62, studies in various fish species have consistently demonstrated that transcripts and proteins, corresponding to expression of the *cyp19a1b* gene (aromatase B), were exclusively detected in radial glial cells 26,41,43,53,63–66. Such cells play a major role in brain development because they are the origin, either directly or undirectly, of all brain cell types, including neurones and astrocytes 67,68, and this is also the case in adult fishes 41. Radial glial cells largely persist in the brain of adult teleost fishes in which they continue to generate new neurones throughout life 41,69–72. Until now, the functional significance of the strong expression of aromatase in the brain of fish, and specifically in radial glial cells, has not been understood. In the *X. laevis* brain, using radial glial, proliferation and neuronal markers, we demonstrate that *cyp19a1* was probably exclusively expressed in neurones at all of the developmental stages studied, including post-metamorphic stages (juvenile and adult). Indeed, we demonstrated that radial glial cells and neural progenitors do not express *cyp19a1*. As noted above, under normal conditions, aromatase expression in the brain was largely reported in neurones, notably in the telencephalon and diencephalon of mammals 60,61,73 and birds 74,75, and in a very limited number of studies, in astrocytes 76,77. In summary, under normal conditions, *cyp19a1* expression in amphibian *X. laevis* is very similar to other vertebrates in terms of brain aromatase-positive areas. Regarding the cell types expressing the aromatase enzyme encoding gene, our data suggest that, similar to mammals and birds, *cyp19a1* expression is restricted to neurones. This is in sharp contrast with the situation in fish where aromatase is only found in radial glial cells of the developing and adult fish brain. Interestingly, in birds and mammals, reactive astrocytes express aromatase following brain injury and ischaemia 21,22,78–80. Because amphibians are well known for the high capability of their brains to regenerate after injury 81,82, it would be interesting to investigate whether ectopic *cyp19a1* expression could be detected in radial glial cells in amphibians during the regenerative process, in a similar way to that occurring in other vertebrates.

In conclusion, the present study fills a gap in the literature regarding *in situ cyp19a1*/aromatase expression in vertebrates by reporting information on the detailed sites of the expression of *cyp19a1* during brain development in the amphibian *X. laevis*. We show that, similar to other tetrapods, *cyp19a1* expression is mainly found in neurones, in contrast to the situation in teleosts. Although the present study represents a significant advance in our knowledge regarding *cyp19a1* expression in the developing brain of the *Xenopus*, there are still many issues that remain to be adressed. In particular, it would be of interest to document the potential sexually dimorphic expression of *cyp19a1* in the brain regions expressing this gene in adults. It would also be informative to compare *in situ cyp19a1* expression with that of oestrogens and androgen receptors during brain development and adults. In teleosts, it is well documented that *cyp19a1b* expression is strongly regulated by oestrogens and some androgens, making this gene a target for xeno-oestrogens 23,63,83. Whether this is also the case in amphibians requires further investigation given the sensitivity of amphibians to endocrine disruptors 84.
